# A membrane-based microfluidic device for mechano-chemical cell manipulation

**DOI:** 10.1007/s10544-016-0040-8

**Published:** 2016-03-03

**Authors:** Agnese Ravetto, Imo E. Hoefer, Jaap M. J. den Toonder, Carlijn V. C. Bouten

**Affiliations:** Department of Biomedical Engineering, Eindhoven University of Technology, Eindhoven, Netherlands; Deparment of Experimental Cardiology, University Medical Center Utrecht, Utrecht, Netherlands; Department of Mechanical Engineering, Eindhoven University of Technology, Eindhoven, Netherlands; Institute for Complex Molecular Systems, Eindhoven University of Technology, Eindhoven, Netherlands

**Keywords:** Mechanical deformation, chemical stimulation, integrated membrane, circulating cell mechanics

## Abstract

We introduce a microfluidic device for chemical manipulation and mechanical investigation of circulating cells. The device consists of two crossing microfluidic channels separated by a porous membrane. A chemical compound is flown through the upper “stimulus channel”, which diffuses through the membrane into the lower “cell analysis channel”, in which cells are mechanically deformed in two sequential narrow constrictions, one before and one after crossing the stimulus channel. Thus, this system permits to measure cell deformability before and after chemical cues are delivered to the cells within one single chip. The validity of the device was tested with monocytic cells stimulated with an actin-disrupting agent (Cytochalasin-D). Furthermore, as proof of principle of the device application, the effect of an anti-inflammatory drug (Pentoxifylline) was tested on monocytic cells activated with Lipopolysaccharides and on monocytes from patients affected by atherosclerosis. The results show that the system can detect differences in cell mechanical deformation after chemical cues are delivered to the cells through the porous membrane. Diffusion of Cytochalasin-D resulted in a considerable decrease in entry time in the narrow constriction and an evident increase in the velocity within the constriction. Pentoxifylline showed to decrease the entry time but not to affect the transit time within the constriction for monocytic cells. Monocytes from patients affected by atherosclerosis were difficult to test in the device due to increased adhesion to the walls of the microfluidic channel. Overall, this analysis shows that the device has potential applications as a cellular assay for analyzing cell-drug interaction.

## Introduction

Blood cell deformability can affect vascular flow and can play a significant role in cardiovascular diseases, such as chronic inflammatory diseases. Drugs used for vascular complications can change biophysical properties of blood cells, due to their effect on cell membrane, cell cytoskeleton and cell fate, such as apoptosis (Lam et al. 2007; Ruef et al. 2010; Rosenbluth et al. 2008; Wuang et al. 2011; Dai et al. 2010). Therefore, it is of significant importance to study mechano-biological changes of diseased circulating cells in the development of a new treatment.

The analysis of mechanical effects of cell-drug interaction is essential for biochemistry and clinical applications, for example in drug screening and toxicity testing. Despite the fact that conventional macroscopic assays for drug development are still widely used, microfluidic technology has attracted increasing interest in the past years (Nguyen et al. 2013; Abkarian et al. 2006; Dai et al. 2010; Adamo et al. 2012; Mao et al. 2012; Ma et al. 2009). Integrated microfluidic devices can be used to perform reproducible analyses on small sample volumes, at reduced reagent consumption, and with precise system control. Furthermore, microfluidics is being regarded as an excellent platform for cell-based assays since it enables analysis at the single cell level in an *in vivo*-like environment, allowing for controlled cell and biomolecule manipulation and interrogation, while still offering the perspective to analyze many cells within a short time. In particular, controlling diffusion of chemical agents in a microfluidic device permits to realize a stable chemical gradient that can be maintained for prolonged periods of time, or create stable concentration levels of chemicals relevant for cellular assays. At the same time, microfluidic devices offer the ability to observe in real-time the cellular responses to applied chemicals. Thus, microfluidic-based cell platforms can be used to generate biomolecular gradients mimicking biological signaling occurring *in vivo*, and to test concentration-dependent cellular response to chemical treatments.

Recently, a number of cell-based microfluidic systems have been described that have a perfusion cell culture chamber in which medium flow is not only used to feed cultured cells continuously, but also to provide additional functionalities, such as generating gradients of drug concentrations. Liu et al. developed a microfluidic method for monitoring in real time the effect of an anticancer drug on cancer glioma cells (Liu et al. 2010). Furthermore, to generate stable chemical gradients without producing shear stress over cultured cells, gradient barriers such as membranes (Abhyankar et al. 2006; Kim et al. 2009) or hydrogels (Saadi et al. 2007; Cheng et al. 2007; Mosadegh et al. 2007) have been integrated in microfluidic devices. These systems allow establishing a controllable chemical gradient without affecting the fluid flow in the cell area. Kim et al. (Kim et al. 2009) developed a device that generates a steep gradient interface of small molecules over a cell culture. They established the chemical gradient by separating the gradient channel and the cell culture channel with a polyester membrane and measured the uptake of the chemical by the cultured cells. Abhyankar et al. (Abhyankar et al. 2006) created a stable chemical gradient without requiring fluid flow by diffusion through a membrane from a chemical source region. Saadi et al. (Saadi et al. 2007) developed a microfluidic device for generating stable concentration gradients based on a two-compartment diffusion system consisting of two large parallel channels connected by horizontal microgrooves filled with collagen gel. The device was validated by observing neutrophil chemotaxis in a soluble chemoattractant (IL-8) gradient. Cheng et al. (Cheng et al. 2007) used a hydrogel-based microfluidic device to monitor cell migration and chemotactic response of suspension cells.

Another benefit of microfluidic devices is that it is possible to accomplish sequential processes in a single device, such as sample handling, controllable mixing and diffusion of chemicals, detection and analysis. The design of the system can then be integrated with additional features to achieve multiple measurements in a single experiment.

**Fig. 1:**
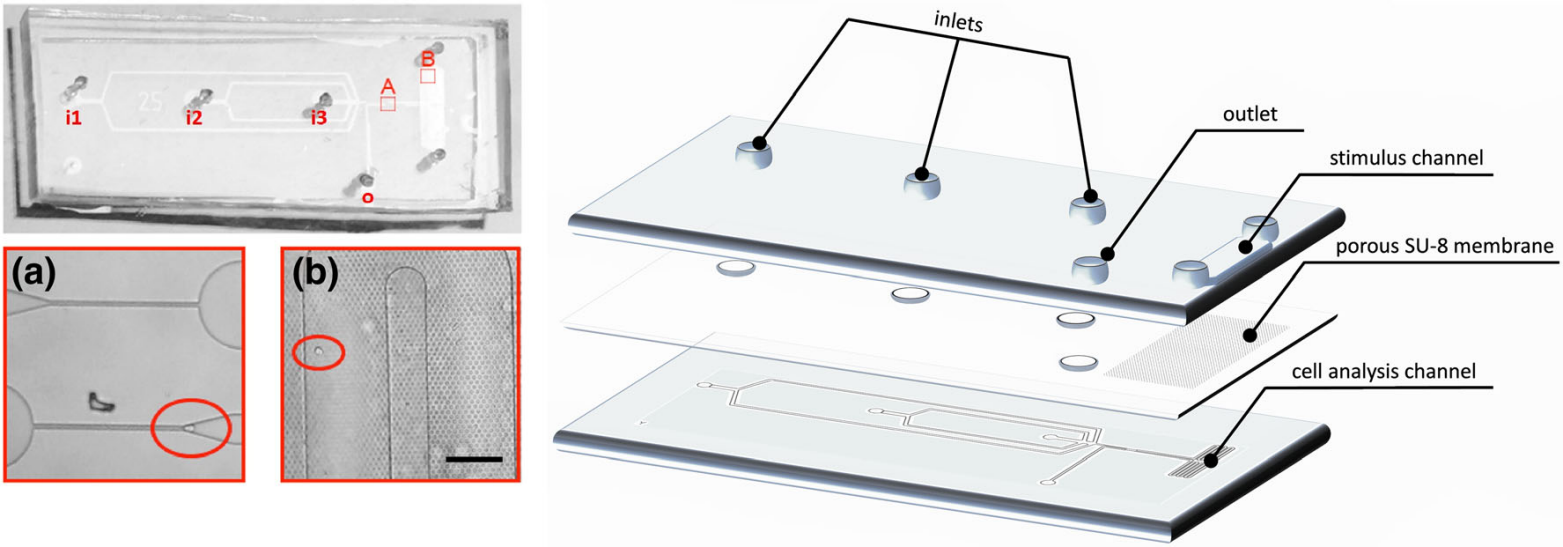
*Left*, Images of the microfluidic device consisting of three inlet holes (i1, i2, i3), two narrow constrictions (**a**), the serpentine channel with the upper stimuli channel (**b**) and the outlet hole (o). The magnified images show (**a**) the two constrictions with a cell entering in the first channel (*red circle*) and (**b**) the serpentine with the porous membrane on top, with a flowing cell stimulated by the chemical compound diffusing through the membrane from the upper stimulus channel. Scale bar for magnified images A&B is 100 μm. *Right*, 3D exploded view of the device displaying the three components of the device (the upper stimulus channel, the porous membrane and the lower cell analysis channel with two sequential constrictions)

In this paper, we discuss the design, the fabrication and the characterization of a new microfluidic device that integrates two single-cell functions, namely mechanical and chemical manipulation. The drug stimulation chip developed in this study has the potential to be used for the analysis of cell-drug interaction due to the ability to test the mechanical effects of chemicals in a single chip. This device consists of two crossing channels separated by a porous membrane (Fig. 1): the chemical compound flows in the upper channel and can diffuse through the membrane to the lower channel where the cells flow and in which the mechanical cell analysis takes place. This cell analysis channel consists of two sequential constrictions, one before and one after a serpentine structure at which the two channels cross and where the chemical stimulation of the cells takes place. The constrictions were designed to investigate cell stiffness in correlation to its influence on cell trafficking through the channels before and after chemical stimulation. Hence, in one single device, the serpentine enables cell retention for drug delivery to the cells through the porous membrane connected to an upper stimuli channel, and the constrictions enable mechanical characterization before and after the drug delivery.

In order to validate the device, the effect on mechanical properties of circulating cells of Cytochalasin-D, an actin-disrupting chemical, was investigated. Furthermore, the effect of Pentoxifylline, an anti-inflammatory drug, both on LPS-activated monocytic cells and on monocytes from patients affected by atherosclerosis was testes as a demonstration of the application of the device. Treatment with Cytochalasin-D resulted in a considerable decrease in entry time in the constriction and an evident increase in the velocity within the constriction. Application of Pentoxifylline to LPS-activated cells resulted in a slight decrease of entry time but no relevant change in the speed in the narrow channel was observed. Analysis of monocytes from patients affected by atherosclerosis resulted extremely difficult due to increased adhesion of flowing cells to the walls of the microfluidic channel. Overall, the results demonstrate the feasibility of the system for simultaneous chemical manipulation and analysis of cellular mechanical changes.

## Materials and methods

### Device principle

The constriction design permits the investigation of cell stiffness, while chemical diffusion through a porous membrane enables chemical manipulation.

Constricted microchannels have been used previously in microfluidic analysis of cell mechanical properties. Since this structure geometrically mimics the *in vivo* capillary-like microenvironment, it allows for mimicking the biorheological behavior of cells as they pass through narrow constrictions of the blood capillaries. Constriction channels, which are smaller than the diameters of tested cells, provide an effective method to generate mechanical stimuli. Multiple parameters, such as entry time, transit time, elongation and recovery time, in association with cell deformability, can be quantified.

The integration of porous membranes into microfluidic devices offers many opportunities, such as diffusion of chemicals between two channels or chambers. The diffusion of chemicals through the porous membrane integrated in our chip depends on the difference in concentration between the upper stimulus channel and the lower analysis channel. The diffusion *D* of the chemical compound is defined by the Stokes-Einstein equation (Wijmans and Baker 1995; Mehta and Zydney 2005)

1 $$ D=\frac{KT}{6\pi R\eta }, $$

where *K* is Boltzmann’s constant, *T* the temperature, *η* the fluid viscosity, and *R* the molecule radius. By substituting the estimation of the molecule radius, the diffusion *D* results

2 $$ D=\frac{KT}{6\pi \eta }{\left(\frac{4\pi \rho N}{3M}\right)}^{\frac{1}{3}}, $$

where *ρ* is the fluid density, *N* the Avogadro number and *M* the molecular weight of the diffusing molecule. Then, the flux *J* through the membrane reads

3 $$ \mathbf{J}=nD\nabla C=n\frac{KT}{6\pi R\eta }{\left(\frac{4\pi \rho N}{3M}\right)}^{\frac{1}{3}}\nabla C, $$

where *n* is the porosity of the membrane and ∇*C* is the concentration gradient.

In our device the length of the serpentine channel is designed to be much larger than the diffusion length, defined as the distance that the compound travels by diffusion while being transported by the fluid flow at the imposed flow rate through the serpentine channel. This design of the serpentine channel allows the compound to get into contact with the flowing cells in the lower microfluidic channel for the desired residence time.

The height of the microfluidic analysis channel and of the stimuli channel was 20 μm. The constriction channel had a width of 7.5 μm and a length of 250 μm. The serpentine channel had a width of 150 μm and a length of 31 mm. The stimuli channel had a width of 2.4 mm and a length of 7.5 mm.

The fluid flow was driven by applying a hydrostatic pressure drop over the device. The pressure drop was generated by the difference in height of a liquid in reservoirs in the inlet and the outlet. By carefully adjusting the liquid levels, the liquid flow rate can be regulated. The flow rate was adjusted to obtain sufficient incubation time of the cells with the drug while flowing through the serpentine. The pressure drop is given by

4 $$ \varDelta p=QR=\left(\frac{L}{t}A\right)\left(\frac{12\mu L}{w{h}^3\left(1-0.63\frac{h}{w}\right)}\right), $$

where *Q* is the flow rate and *R* is the hydraulic resistance, *t* is the mean fluid residence time within the channel, *μ* is the dynamic viscosity. *L*, *w*, *h*, *A a*re the geometrical characteristics of the channel (length, width, height and area respectively). The cell residence time was measured also experimentally within the microfluidic serpentine, by recording the cell trafficking through the complete serpentine. In these preliminary studies, the effect of deformation in the first constriction on cell mechanical properties was considered by observing the difference in entry time and cell velocity between the first and second constrictions.

### Design and fabrication

**Fig. 2:**
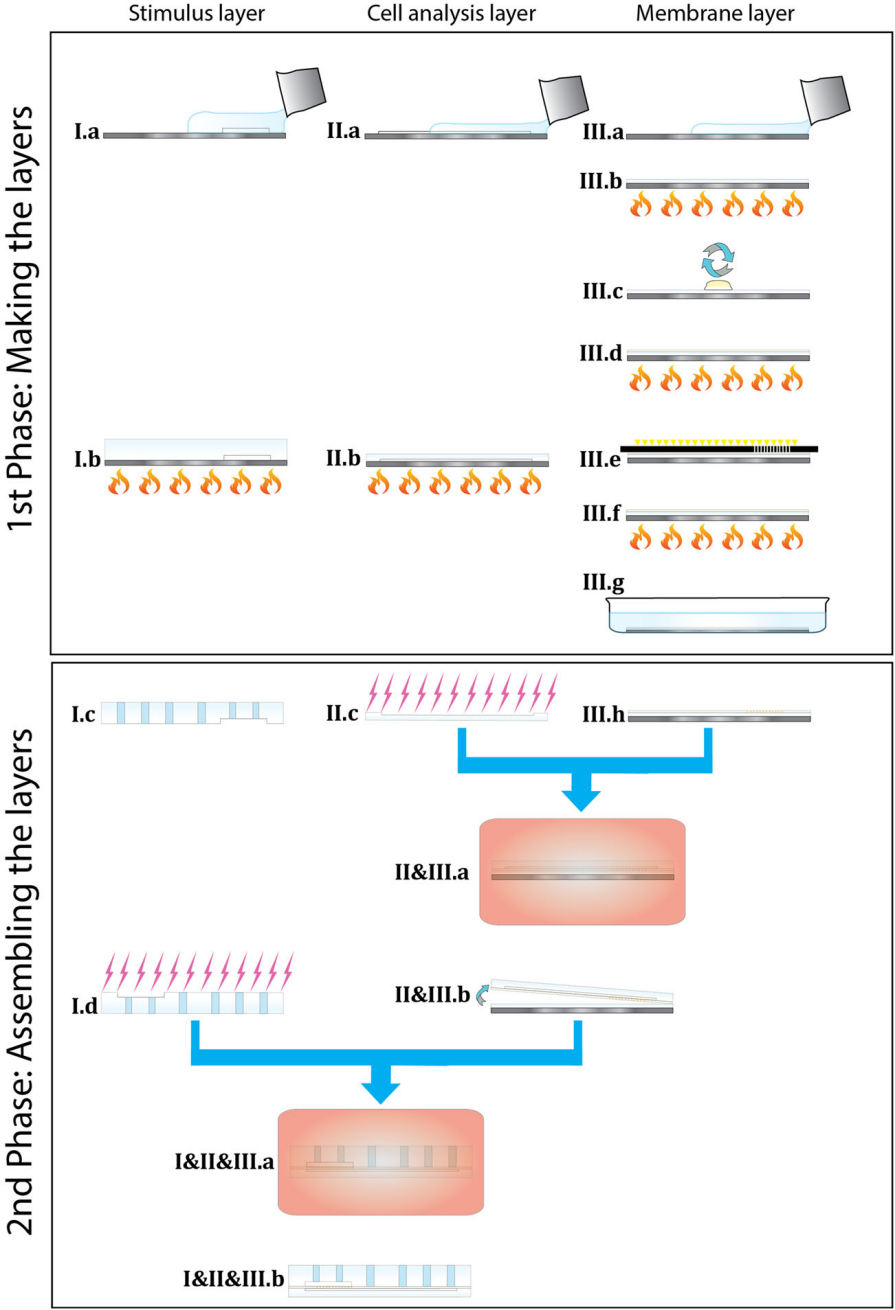
Steps of the fabrication of the microfluidic device, divided in a 1st Phase: Making the layers and a 2nd Phase: Assembling the layers. First, for the fabrication (1st Phase), the stimulus (I) and the cell analysis (II) layers were fabricated by pouring PDMS on the microstructures of the master (I.a and II.a) and by curing the elastomer for 1 h at 110 °C (I.b and II.b). The membrane layer (III) was fabricated by photolithography of an SU-8 2010 film on a PDMS layer. The PDMS mixture was poured on a silicon wafer (III.a) and was cured (III.b). SU-8 2010 was spun onto the PDMS layer (III.c) and it was pre-baked (III.d) with a controlled temperature ramp. Then, the layer was exposed by UV light through a mask defining the porous structure (III.e), post-baked (III.f) and then developed in SU-8 developer (III.g). For assembling the device (2nd Phase), the PDMS cell analysis layer was activated in nitrogen plasma (II.c) and immediately aligned and bonded to the SU-8 membrane layer and baked overnight (II&III.a). The SU8-PDMS structure was then peeled off from the PDMS sacrificial layer (II&III.b). Inlet and outlet ports were cut in the stimulus PDMS layer (I.c). The stimulus layer was then aligned to the SU-8 membrane and bonded by nitrogen surface treatment (I.d) followed by curing overnight (I&II&III.a) to get the final assembled device

The device consisted of three components (Fig. 1): the upper stimulus channel with inlets and outlet holes (Fig. 2, I), the cell analysis channel (Fig. 2, II) and the porous membrane located between these two channels (Fig. 2, III). The device was fabricated from two layers of (poly)dimethylsiloxane (Sylgard 184, Dow Corning, The Netherlands), for the upper stimulus channel (I) and for the lower cell analysis channel (II), and a custom-made SU-8 membrane with through pores 3 μm in diameter (III).

The fabrication process is shown in Fig. 2 as two phases, namely the 1st Phase illustrating the fabrication of the three layers (two PDMS layers and one SU-8 membrane layer) and the 2nd Phase illustrating the assembly of the layers. Briefly, the PDMS layers (I&II) were fabricated using prototyping and multilayer soft lithography. The stimulus layer (I) and the cell analysis layer (II) were generated from two transparency masks made in plastic foil and chromium on glass, respectively. The photoresist-based negative molds (SU-8, MicroChem, Germany) were patterned on silicon wafers from the transparency masks by contact photolithography. Positive replicas were fabricated by pouring a 10:1 mixture of PDMS on the microstructures of the master of the stimuli channel and of the cell analysis channel (Fig. 2, I.a and II.a) and by curing the elastomer for 1 h at 110 °C (1st Phase: I.b and II.b).

The membrane layer (III) was fabricated in a unique and custom way by photolithography of an SU-8 2010 film on a PDMS layer used both as carrier and as sacrificial substrate. In detail, the PDMS mixture was poured on a silicon wafer, was allowed to evenly spread on the substrate on a flat surface for 30 min at room temperature (Fig. 2, 1st Phase: III.a) and subsequently it was cured at 110 °C for 1 h (Fig. 2, 1st Phase: III.b). SU-8 2010 was spun onto the PDMS layer at a spinning frequency of 500 rpm for 10 s, followed by a cycle of 3000 rpm for 30 s (Fig. 2, III.c). This spincoating procedure resulted in a 7-μm-thick SU-8 layer. After spinning, the SU-8 layer was pre-baked (Fig. 2, 1st Phase: III.d) with a controlled temperature ramp (first 2 min at 50 °C followed by 10 min at 65 °C). Then, the layer was exposed by UV light (Near UV Exposure System 350 Watt, ABM, USA) with an exposure energy of 120 mJ/cm^2^ through a mask defining the porous structure (Fig. 2, 1st Phase:III.e). Finally, the SU-8 layer was post-baked for 3 min at 90 °C (Fig. 2, 1st Phase:III.f) and then developed for 2 min in SU-8 developer (MrDev, Mircoresist, Germany) to remove the uncrosslinked resist (Fig. 2, 1st Phase:III.g).

In order to assembly the device, the layers were attached to each other by following these sequential steps (2nd Phase). The PDMS cell analysis layer was activated in nitrogen plasma (Fig. 2, 2nd Phase: II.c) and immediately aligned and bonded to the SU-8 membrane layer; the adhesion was consolidated by baking the structure at 75 °C overnight (Fig. 2, 2nd Phase: II&III.a). The SU8-PDMS structure was then peeled off from the PDMS sacrificial layer (Fig. 2, 2nd Phase: II&III.b). Lastly, the stimulus layer had to be bonded to complete the device. Inlet and outlet ports were punched in the stimulus PDMS layer (Fig. 2, 2nd Phase: I.c) using a biopsy needle (Harris, The Netherlands). The stimulus layer was then treated by nitrogen plasma (Fig. 2, 2nd Phase: I.d) and subsequently aligned and attached to the SU-8 membrane followed by curing at 75 °C overnight (Fig. 2, 2nd Phase: I&II&III.a). The final assembled device (Fig. 2, 2nd Phase: I&II&III.b) was connected by silicon tubing to liquid reservoirs to generate the desired flow in the channels driven by hydrostatic pressure difference. Prior to cell analysis, the channel was coated with 1 % non-adhesive Pluronic solution for 1 h and washed with phosphate buffered saline for 40 min.

### Cell culture and chemical stimulation

Human promyelocytic leukemia HL60 cells were used as a model cell line for monocytes. The cells were obtained from the European Collection of Cell Cultures (ECACC) and cultured in RPMI 1640 medium (Invitrogen, The Netherlands) with 2 mM L-glutamine (Lonza, Belgium) and 10 % Fetal Bovine Serum (Greiner, The Netherlands). Cell cultures were maintained at a concentration between 1−9 × 10^5^ cells/ml at 37 °C in a humidified atmosphere containing 5 % CO_2_. Cell differentiation towards monocytic lineage was stimulated by treatment with 0.4 mM Sodium Butyrate (Sigma, The Netherlands), for 4 days at culturing conditions prior to experiment. Cell activation was induced by incubation with 2 μg/ml Lipopolysaccharides (LPS, Sigma, The Netherlands) for 15 min at 37 °C.

For the validation experiment, non-treated HL60 cells (NT) were stimulated by diffusion of Cytochalasin-D (CytoD, Sigma, The Netherlands) through the porous membrane from the upper stimulus channel to the lower cell channel. For the demonstration of the application of the device, both activated cells (LPS) and monocytes from patients affected by atherosclerosis were stimulated by diffusion of Pentoxyfilline (PTX) through the porous membrane.

### Data analysis

An inverted microscope (Zeiss Observer Z1) equipped with a high-speed camera (MotionScope M5, Idtvision) was used to record cell trafficking in the microfluidic channel.

Cell entry time was defined as the time between the cell getting across the entry of the constriction and the cell clearing the entrance. Cell velocity was calculated by using the time interval between the cell clearing the entry and the cell reaching the end of the constriction. Cell entry time and velocity in the channel were plotted against cell diameter. Cell entry time and cell velocity in the channel were measured from recorded videos, both for the first (before chemical stimuli, NT cell number = 31, LPS cell number = 19) and for the second constrictions (after chemical stimuli, CytoD cell number = 41, PTX cell number = 15).

The relationships between trafficking parameters and cell diameter were analysed with linear regression and the statistical significance of the differences in entry time and velocity in the channel were tested by comparing the regression line slopes and the intercepts (with values of *p* < 0.05 considered significant).

## Results

### Experimental design

The concentration of the chemical in the upper chamber was calculated based on Eq. 3, in order to reach in the lower cell analysis channel a concentration of the compounds used previously in literature (Ruef et al. 2010; Du et al. 2011) within the first minute of experimentation. For Cytochalasin-D, a concentration of 5 μg/ml was used and for PTX a concentration of 1 mg/ml was chosen.

Furthermore, the flow rate in the cell analysis channel generated by the hydrostatic pressure drop was calculated both theoretically by Eq. 4 as well as determined experimentally. For the experiment with Cytochalasin-D a difference in height of 25 mm corresponds to a cell residence time of 10 min in the serpentine (corresponding to a flow rate of around 10 nl/min). For the experiment with PTX a height difference of 50 mm was used in order to stimulate the cells for 5 min in the serpentine (around 19 nl/min).

In preliminary experiments, while flowing only the buffer in the upper stimulus channel, we observed that cells recovered completely in the serpentine channel from the deformation in the first constriction, and that, upon complete cell recovery, entry time and cell velocity in the second constriction were not affected by cell transit in the first constriction at any analyzed flow rates.

### Validation of the device

CytoD, a known actin-disrupting agent, was used to demonstrate the working principle of the device. The HL60 cell solution was added in the lower microfluidic channel while the CytoD solution was added in the upper stimulus channel.

**Fig. 3:**
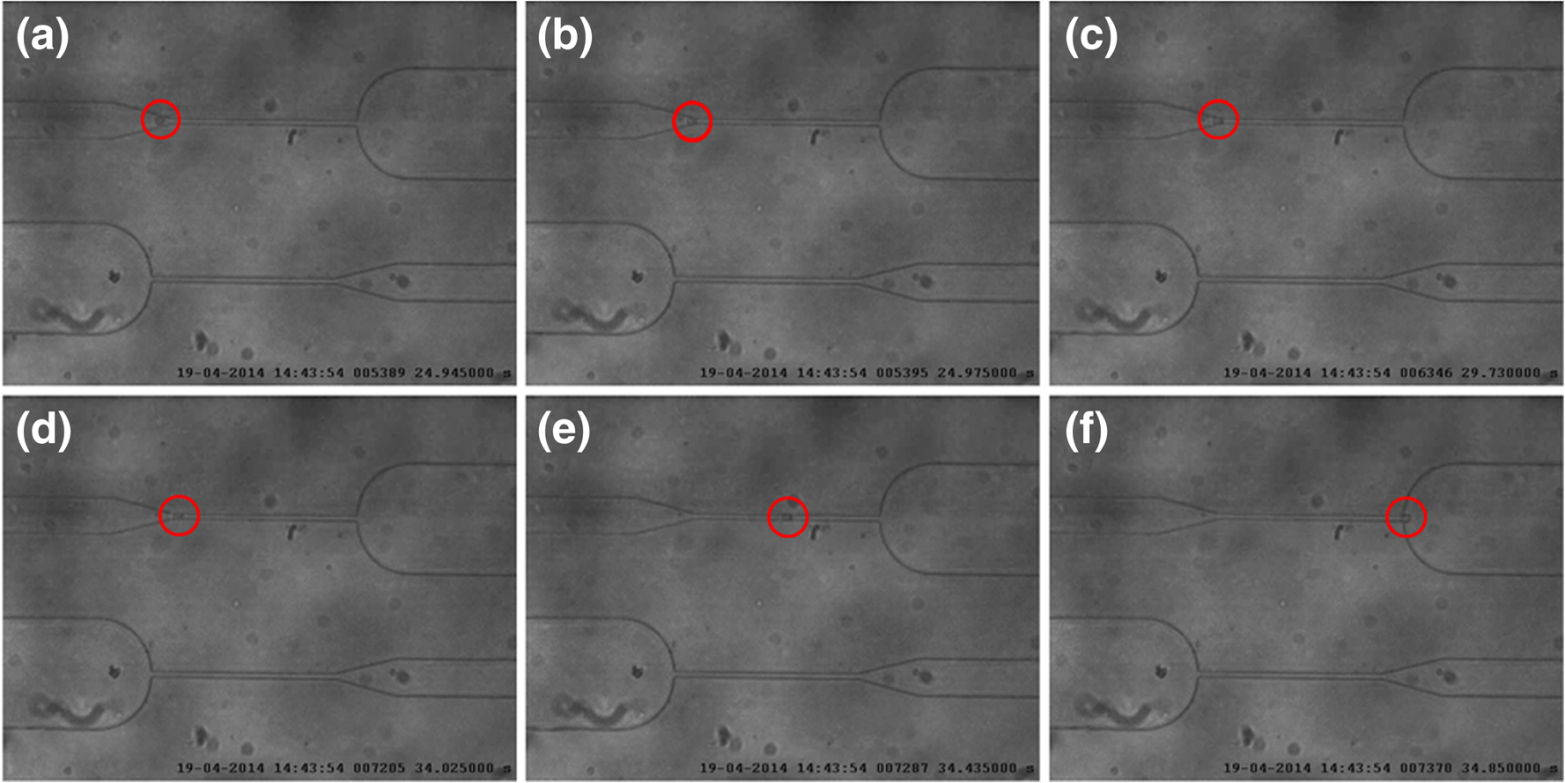
Images captured from the video taken of a HL60 cell flowing in the first constriction (before Cytochalasin-D stimulation). The entry time (interval B-D) is around 10 s (from 24.975 to 34.025 s). The trafficking time (interval D-F) is 0.83 s

**Fig. 4:**
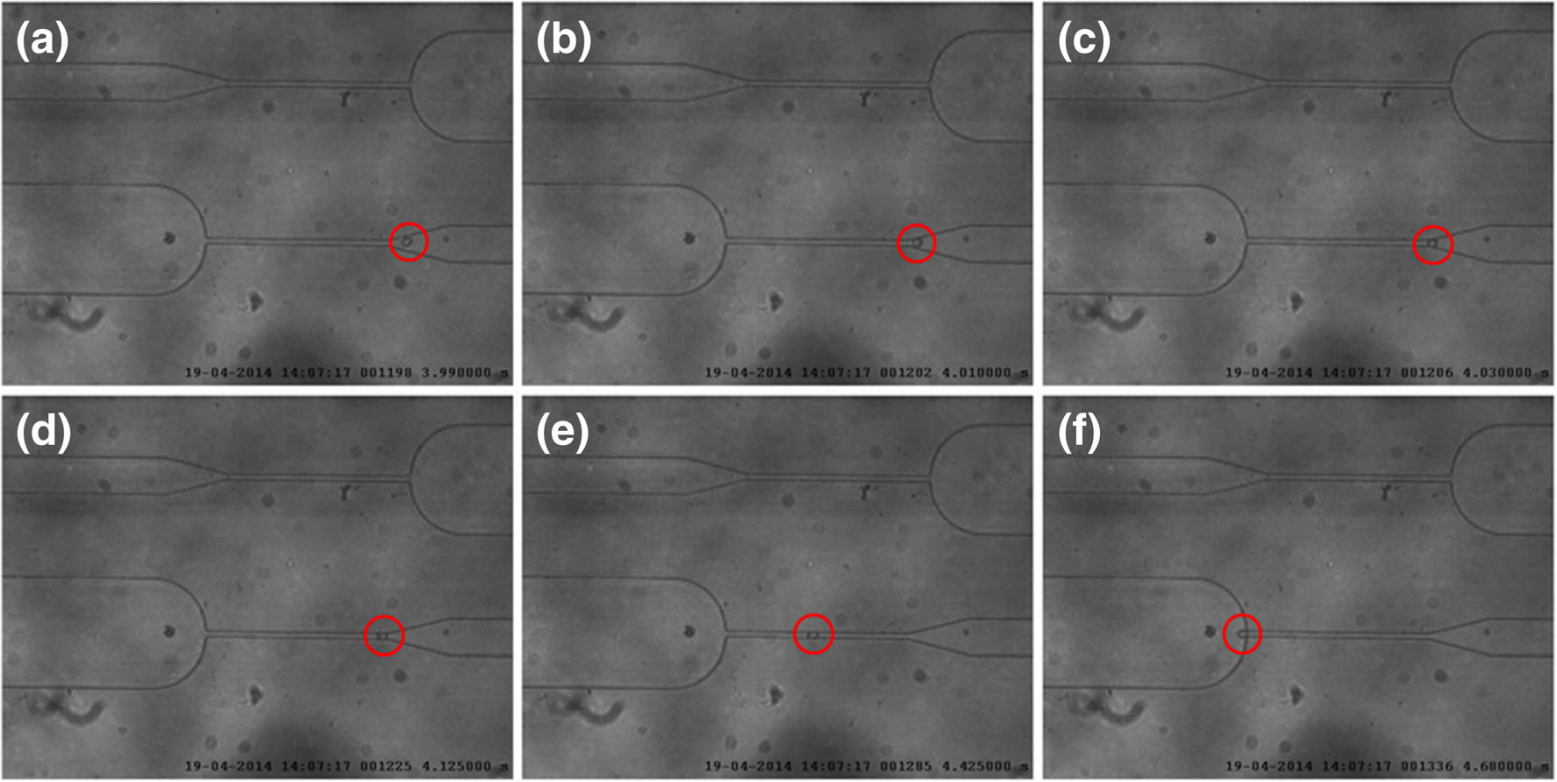
Images captured from the video of an HL60 cell flowing in the second constriction (after Cytochalasin-D stimulation through the porous membrane). The entry time (interval B-D) is around 0,11 s (from 4010 to 4.125 s). The trafficking time (interval D-F) is 0.55 s

**Fig. 5:**
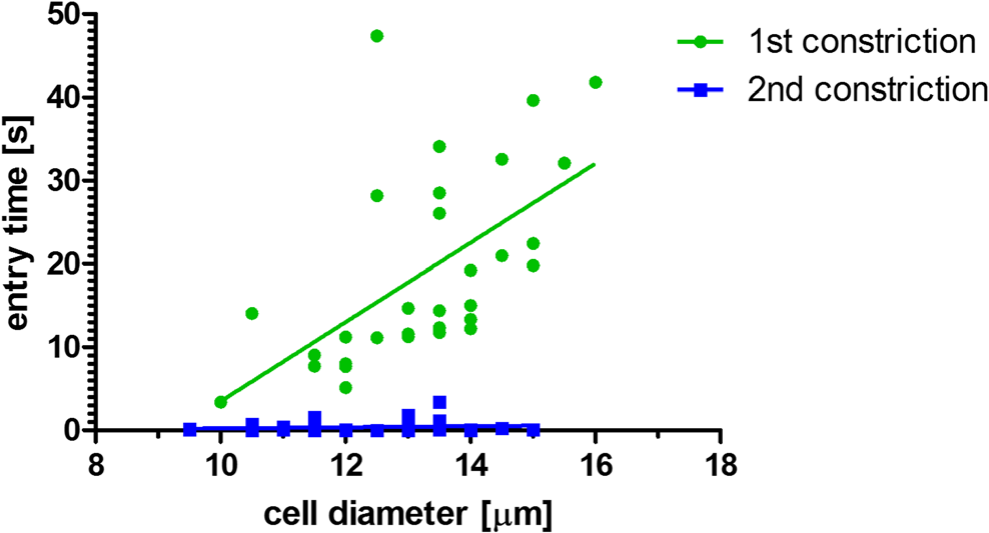
Entry time of HL 60 cells in the first constriction (before CytoD stimulation, *green circles*) and in the second constriction (after CytoD stimulation through the porous membrane, *blue squares*). The entry time is plotted as a function of cell diameter. There is a clear dependency on cell diameter for non-treated cells, while this dependency is not present for CytoD-treated cells. Most importantly, the entry time is substantially shorter after CytoD stimulation. The data were fitted by a linear regression line (for the first constriction R^2^ = 0.34 and for the second constriction R^2^ = 0.02)

**Fig. 6:**
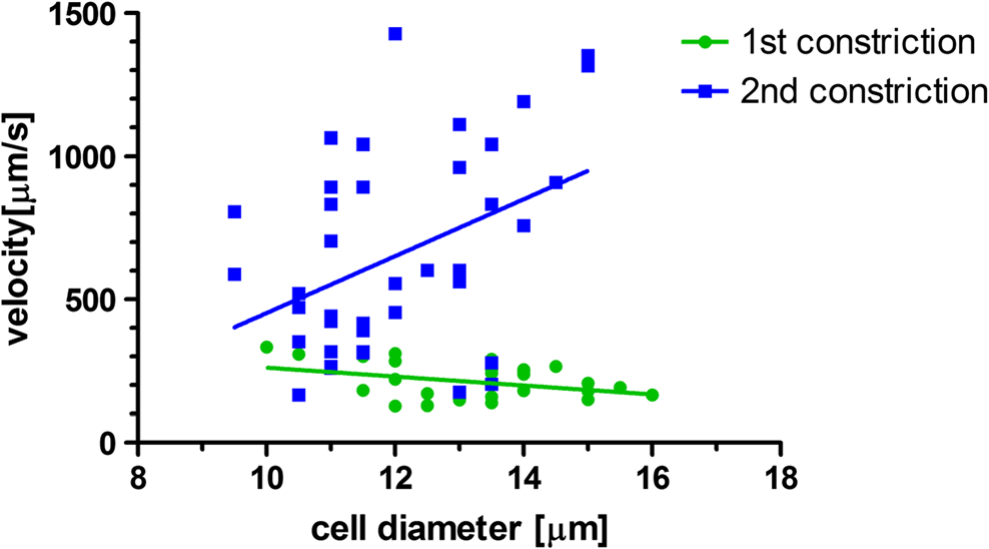
HL60 cell velocity in the first constriction (before CytoD stimulation, *green circles*) and in the second constriction (after CytoD stimulation through the porous membrane, *blue squares*). The velocity is plotted as a function of cell diameter. The trafficking velocity is, overall, higher after treatment with CytoD through the porous membrane. The data were fitted by a linear regression line (for the first constriction R^2^ = 0.13 and for the second constriction R^2^ = 0.17)

Figures 3 and 4 show images captured from videos of HL60 cells flowing in the first and second constrictions, respectively. A difference in trafficking behavior before and after CytoD stimulation can be clearly appreciated from the timing indicated in the images. The entry time (Fig. 5) and speed or trafficking velocity in the channel (Fig. 6) were then calculated as a function of cell diameter. Although the spread in the data is considerable, Fig. 5 clearly shows that the entry time increased with cell diameter for non-treated HL60 cells, while there was no clear diameter dependence for CytoD stimulated cells. Most importantly, there was a strong decrease in the time required to enter in the confined channel after stimulation of the cells by CytoD. Although the spread of the data is relatively high, as evidenced by the low R^2^ value, the differences between the slopes are significant.

**Fig. 7:**
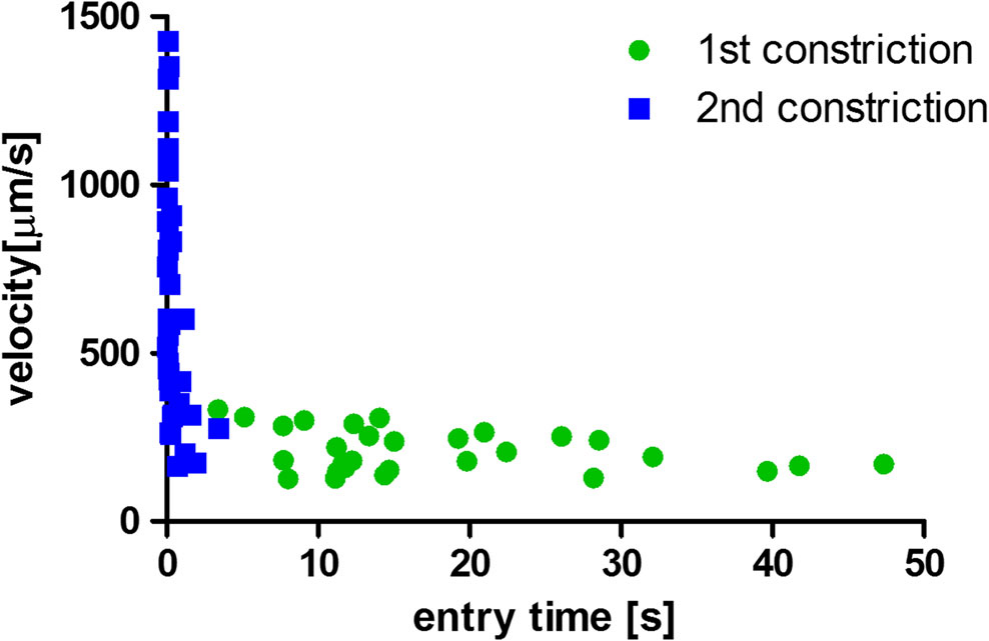
Correlation between cell entry time and cell velocity in the constriction channels. There is a strong distinction between the cells in the first constriction (before the porous membrane, NT HL60, *green circles*) and the cells in the second constriction (after the porous membrane CytoD treated HL60, *blue squares*)

The change in cell trafficking velocity in the constriction due to the CytoD treatment was less evident than for the entry time, as can be seen in Fig. 6. The behavior of non-treated cells depended on cell diameter, with a decrease in speed within the channel as cell diameter increased. The data for CytoD stimulated cells showed too much variation to recognize a clear trend. The spread of data was high and the linear fitting was characterized by an extremely low R^2^ value. This resulted in a contradictory apparent increasing trend in the cell velocity as the cell diameter increased. Nevertheless, difference in cell speed in the constricted channel between the first and the second constriction was observed: cells in the second constriction, after treatment with CytoD in the serpentine section with the porous membrane, had a higher trafficking velocity. The differences between the slopes are significant. Although both the entry time and the transit time are governed by cell mechanical stiffness, the two quantities were measured independently and they were not derivable from each other. The correlation between these two parameters is illustrated in Fig. 7. There is a clear distinction between the measured parameters in the first constriction (not treated cells) and in the second constriction (CytoD-treated cells).

This simple demonstration opens the door for future applications where clinical drugs used on circulating cells could be tested to determine the effect on cell mechanical properties.

### Demonstration of the application

Pentoxifylline (PTX), a drug used in inflammatory diseases, was used to demonstrate the working principle of the system by investigating the effect of this drug on LPS-activated monocytic cells and on monocytes from patients affected by atherosclerosis. Cells were flown through the lower microfluidic channel while the PTX solution was added in the upper stimulus channel.

**Fig. 8:**
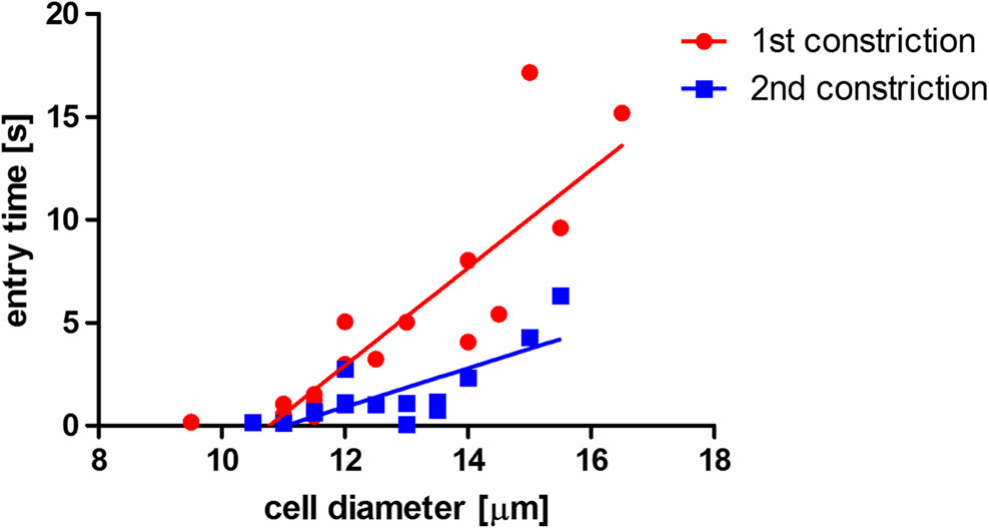
Entry time in the first constriction (LPS-treated HL60 cells before PTX stimulation, *red circles*) and in the second constriction (LPS-treated HL60 cells after PTX stimulation through the porous membrane, *blue squares*). The entry time is plotted as a function of cell diameter. It appears that there is a dependency on cell diameter for both experimental groups, with an increase in entry time for larger cells. The entry time for PTX-treated cells appears slightly smaller than for cells that are only LPS-activated. The data were fitted by a linear regression line (for the first constriction R^2^ = 0.76 and for the second constriction R^2^ = 0.60)

**Fig. 9:**
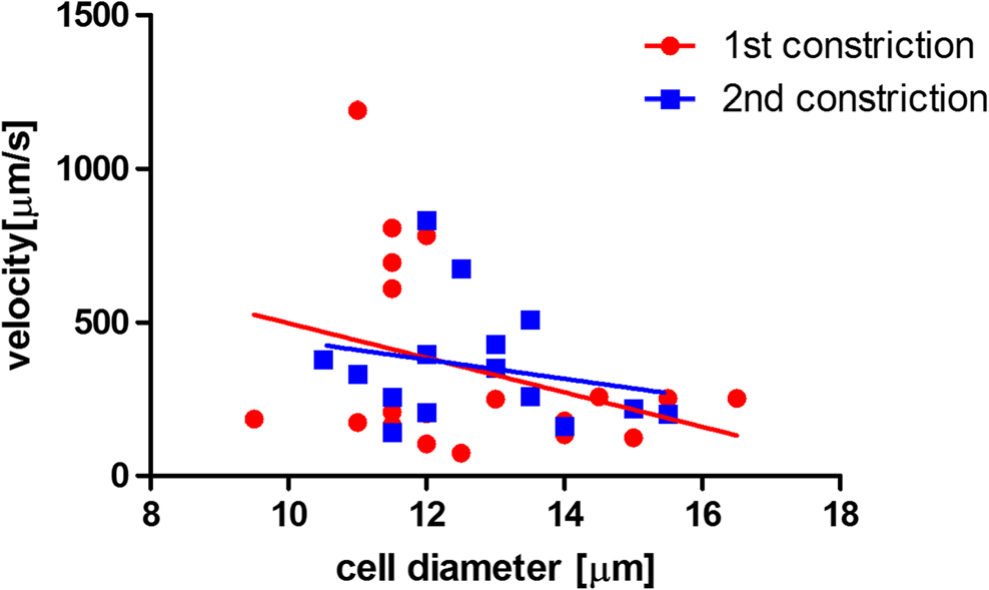
Cell trafficking velocity in the first constriction (LPS-treated HL60 cells before PTX stimulation, *red circles*) and in the second constriction (LPS-treated HL60 cells after PTX stimulation through the porous membrane, *blue squares*). The velocity is plotted as a function of cell diameter. It appears that there is no clear difference in trafficking velocity between the experimental groups. The data were fitted by a linear regression line (for the first constriction R^2^ = 0.11 and for the second constriction R^2^ = 0.05)

Again, the entry time (Fig. 8) and the cell trafficking speed within the constriction (Fig. 9) were determined as a function of cell diameter. There was a clear dependence of the entry time on cell diameter both for LPS activated cells and for activated cells stimulated with PTX. The apparent decrease in entry time after treatment was minor both at small and at large cell diameter. Nevertheless, the differences between the slopes are significant.

The trafficking velocity (Fig. 9) tended to decrease for larger cells, for both cell groups, but the variation in the data was too large, as also evidenced by the low R^2^ value, to draw solid conclusions. Contrary to the CytoD experiment, there was no difference in trafficking velocity in the constriction channel between the two experimental groups. The differences between the slopes and between the intercepts were not significant. Compared to the analysis with CytoD, the differences caused by the drug treatment were not considerable in this case. This might be due to biological reasons, such as the effect of the occasional adhesion of activated cells to the channel wall, or to technical reasons, such as the incapability of the device to detect differences in mechanical properties for these cells and treatment.

**Fig. 10:**
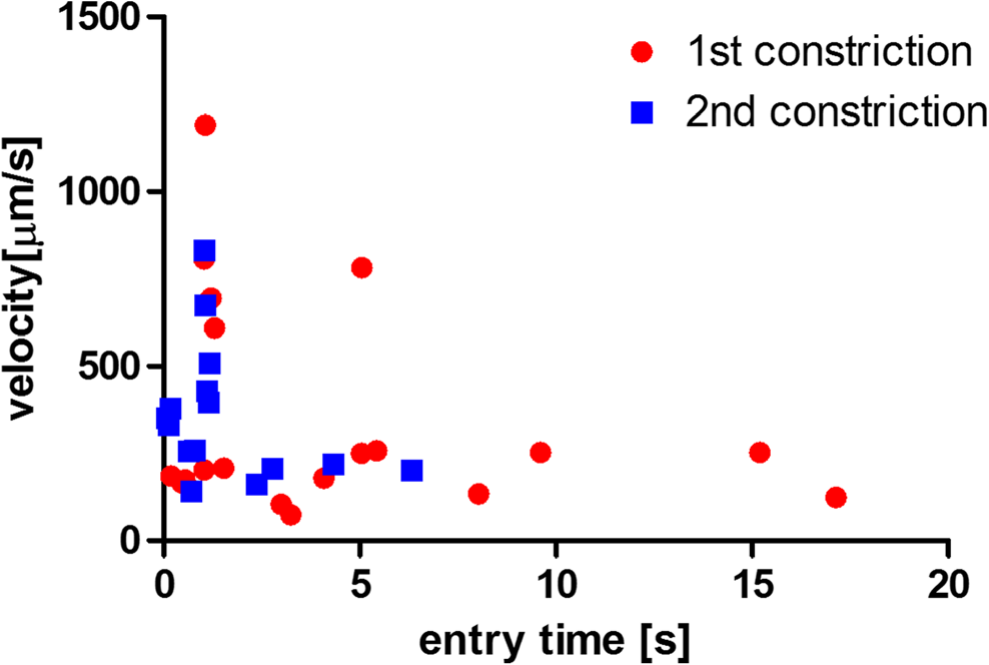
Correlation between HL60 cell entry time and cell velocity in the constriction channels. There is no clear distinction between the cells in the first constriction (LPS-activated only, *red circle*) and the cells in the second constriction (LPS-activated and PTX-treated, *blue squares*)

The correlation between entry time and trafficking velocity in the first and second constrictions is illustrated in Fig. 10. There is no clear distinction between these parameters measured in the two constrictions.

**Fig. 11:**
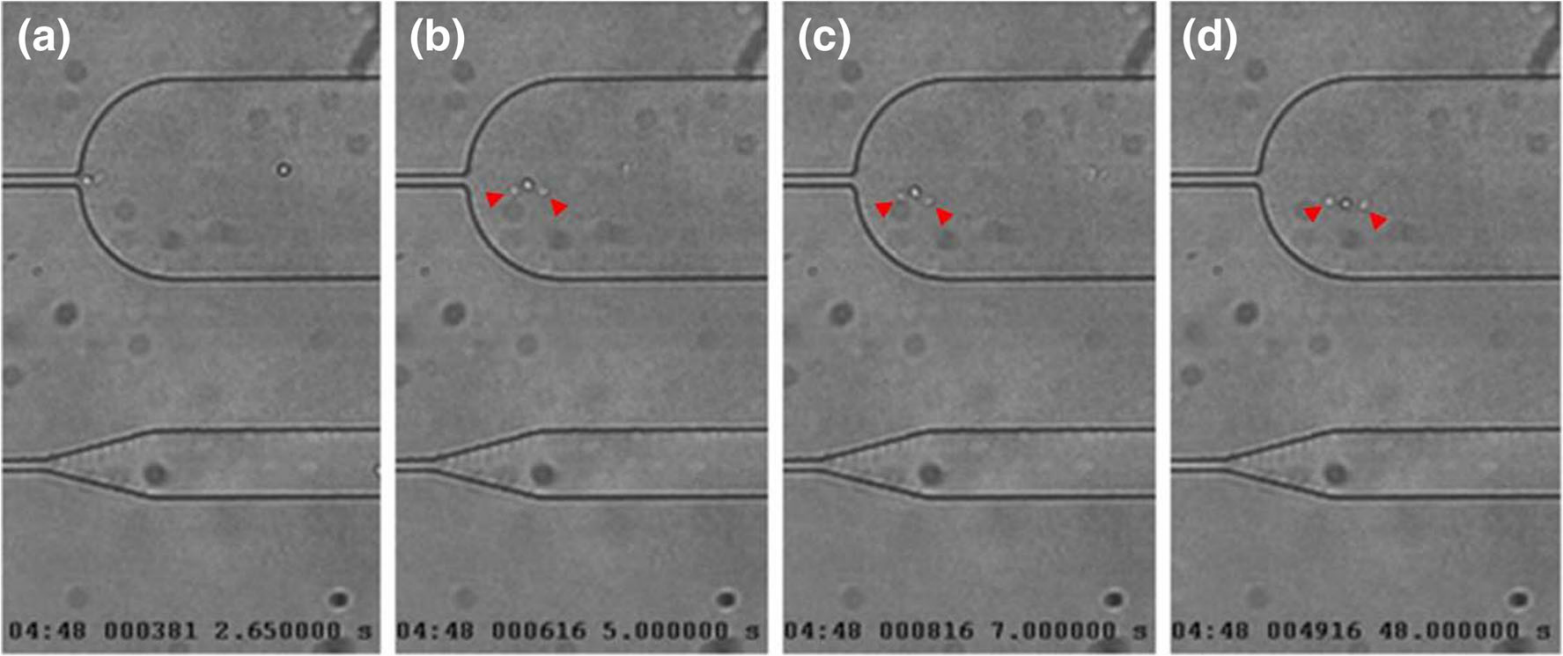
Images captured from a video of a monocyte from an atherosclerotic patient trafficking into a constriction channel. It appears that, while exiting the constriction, the monocyte remained anchored at the channel wall by the projected pseudopods (indicated with *red arrows*)

The atherosclerotic patients’ cells tended to adhere to the walls of the channels and the pseudopods projected by diseased cells where often anchored to the wall of the channel (Fig. 11). Furthermore, since monocytes from atherosclerotic patients tend to form clusters due to increased adhesive properties of cell membrane, it was difficult to investigate the trafficking behavior at single cell level. Thus, cell adhesion interfered with the analysis of cell stiffness since it would influence the entry time and the speed in the channel.

## Discussion and conclusion

Most cellular processes, including those related to diseases, are influenced by both mechanical and biochemical cues and result in changes of the cytoskeletal and mechanical properties of cells. Thus, in the development and testing of cellular assays and disease treatments, it is important to control and manipulate the chemo-mechanical cellular micro-environment and to investigate the resulting cellular response. In this paper, we have introduced and characterized a microfluidic device that permits the investigation of cellular mechanical response to biochemical stimuli at single cell level in a physiologically relevant microenvironment. The key element of the device is a porous membrane that separates a stimulation channel (containing the biochemical agent) and a cell testing channel (through which cells are flown), and through which the agent can diffuse to reach the cells.

Microfluidic systems offer a number of opportunities in the development of drug assays. Several microfluidic systems have been proposed in which membranes or gel layers are used as interfaces through which chemicals can diffuse to the cells (Saadi et al. 2007; Abhyankar et al. 2006; Kim et al. 2009; Cheng et al. 2007). The microfluidic system proposed in this study has the following advantages. First, it requires smaller volumes of stimulating agent compared to conventional chemical assays. The chemical is placed in the stimulus channel of the microfluidic device (with a volume of around 2 μl) and it is allowed to diffuse through the membrane into the lower channel. Furthermore, the diffusion from the upper chamber through a membrane permits a stable chemical transfer without generating fluid shear forces and affecting the flow in the lower analysis chamber.

The fabrication of the device is rather straightforward and reproducible. The device design and the system parameters can be changed to adapt the device to different cellular assays and to increase the sensitivity of the device. Both the stimuli and the cell analysis channels can be modified on the basis of cell type and drug used. For instance, the porosity or the pore size of the membrane could be changed according to the choice of chemical compound and to the desired diffusion rate. The design of the cell microfluidic channel could also be adapted according to the drug used in the assay. For instance, the length of the serpentine channel could be increased if the drug requires a longer time of efficacy. In our validation study we observed a clear distinction in entry time and trafficking velocity between non-treated HL60 cells (first constriction) and CytoD-treated cells (second constriction). This shift in trafficking behavior was not as considerable for LPS-activated cells treated with PTX. Since this result might be due to low sensitivity of the device to smaller changes in cell mechanical properties, the design can be adapted (e.g. by decreasing constriction width) to improve the sensitivity.

On the other hand, the device is not applicable to all circulating cell types and cellular pathologies. Despite the advantages of this technique, cell deformability is associated with cell adhesion between cell membrane and the wall of the channel. Monocytic cells treated with LPS become stickier than non-activated cells. Thus, in the analysis of activated cells, even with the non-adhesive coating pre-treatment of the device, it appeared that the cells tended to stick to the wall or to the floor of the microfluidic channel due to increased adhesive properties of activated cells. For the analysis of activated HL60 cells, the flow rate was increased to avoid cell adhesion on the floor of the serpentine channel. However, this resulted in a decreased sensitivity of the device since the dependency of entry time on cell treatment is enhanced at low pressure differences. Similar behavior was observed for the monocytes from atherosclerotic patients where the cells tended to adhere the pseudopods to the wall of the microfluidic channel. For this reason, the specific design of this setup does not seem to be appropriate for analyses of diseased cells with strong adhesive properties.

Different challenges were encountered in the choice and in the incorporation of the porous membrane in the microfluidic device. The membrane was required to have an array of pores smaller than 5 μm to avoid cell migration towards the upper stimulus channel but at the same time it needed to be fully transparent over the constriction region to avoid the influence of microscopic pores on the observation of cells in the constrictions. Thus, the membrane was custom made as a free-standing SU-8 thin film over a substrate of PDMS as sacrificial layer. The fabrication of the membrane in a clean room has the advantage that any feature can be tailor-made. In fact, photolithography permits straightforward fabrication of micrometer structures according to a preferred design with specific properties and thicknesses. The sealing of membranes in microfluidic devices is a common problem. The incorporation of the membrane was performed at elevated temperature by reaction of the amino groups on the PDMS channel surface, generated by exposure to pure nitrogen plasma, with the epoxy group of the SU8 membrane surface.

The results shown are comparable with previous studies on monocytic cells trafficking through narrow channels after treatment with actin-disrupted agents (Gabriele et al. 2009; Preira et al. 2013). Previous studies on Pentoxifylline showed that this drug reduces pro-inflammatory response and decreases the amount of adhesion proteins, such as ICAM-1, in monocytes (Neuner et al. 1997; Souness et al. 1996). PTX was also shown to have a mechanical effect on blood circulating cells by a significant increase of the deformability of activated granulocytes after 10 min of treatment with a 0.2 mg/ml PTX solution (Ruef et al. 2010). Rosenbluth et al., proposed a microfluidic-based flow cytometer to measure the transit time of HL60 cells stimulated with Cytochalasin D and Pentoxifylline. They observed that median transit time was reduced from 0.63 s to 0.23 s for 1 h exposure of 2 μM of CytoD solution and to 0.37 s for 3 h exposure of 1 mM PTX solution (Rosenbluth et al. 2008).

In our study the change in mechanical properties of LPS-activated monocytic cells after exposure to PTX was not significant. There was a decrease in entry time after stimulation with PTX but no difference was observed in the trafficking velocity within the channel. This could be caused by biological and/or technical reasons. In fact, it can be due to the short residence time in the stimulus region (just 5 min, in contrast to 3 h in Rosenbluth et al.). Furthermore, the measured entry and trafficking time can be affected by the occasional adhesion of activated cells to the wall or to the floor of the channel, as previously described. On the other hand, the increase in the flow rate used in this study could decrease sensitivity of the device, as previously described, or the setup is not sensitive enough to discriminate small differences in mechanical properties. Thus, further investigation is necessary to investigate the sensitivity of the system by varying the driving pressure and by considering different cell types and cellular modifications.

Despite the limitations, the preliminary characterization of our device showed that it has potential applications for various cellular assays investigating the interaction between biochemical and mechanical cues. The device might be adjusted to the use for different applications. For instance, it might be possible to investigate the *in vivo*-like cellular response to molecule release in signaling processes by incorporating the secreted factors in the *in vitro* setup. This might then lead to an enhanced understanding of the biophysical aspects of biological processes, including diseases, and to assess the effect of new treatments on diseased cells.
